# Comparable clinical outcomes but different postoperative Dynamic Alignment of the Knee (DyAK) patterns between cruciate‐retaining and posterior‐stabilized inserts in functionally aligned robotic‐assisted total knee arthroplasty

**DOI:** 10.1002/jeo2.70963

**Published:** 2026-10-28

**Authors:** Christos Koutserimpas, Enejd Veizi, Sébastien Lustig, Konstantinos Dretakis

**Affiliations:** ^1^ School of Health Rehabilitation Sciences University of Patras Patras Greece; ^2^ 2nd Department of Orthopaedic Surgery Hygeia Hospital of Athens Marousi Greece; ^3^ Department of Orthopedic Surgery Aspetar Orthopedic and Sports Medicine Hospital Doha Qatar; ^4^ Department of Orthopedics and Traumatology Ankara Yıldırım Beyazıt University Ankara Turkey; ^5^ Orthopaedics Surgery and Sports Medicine Department FIFA Medical Center of Excellence Croix‐Rousse Hospital, Hospices Civils de Lyon, Lyon North University Hospital Lyon France; ^6^ Université de Lyon, Université Gustave Eiffel, Université Claude Bernard Lyon 1 Lyon France

**Keywords:** Dynamic Alignment of the Knee, functional knee positioning, personalized alignment, robotic knee, TKA

## Abstract

**Purpose:**

The influence of polyethylene insert design during robotic‐assisted total knee arthroplasty (TKA) performed according to functional alignment (FA) principles remains poorly understood. This study compared cruciate‐retaining (CR) and posterior‐stabilized (PS) inserts with respect to implant positioning, clinical outcomes, implant survivorship and postoperative dynamic knee behaviour using the Dynamic Alignment of the Knee (DyAK) classification.

**Methods:**

A retrospective comparative study was performed on 284 primary functionally aligned robotic‐assisted TKAs. The cohort comprised 148 CR and 136 PS fixed‐bearing cemented implants with a minimum follow‐up of 3 years (median 44.5 months). Clinical outcomes included Knee Society Score (KSS) and Forgotten Joint Score (FJS). Intraoperative robotic implant positioning, bone resections, polyethylene thickness, DyAK classification, complications and Kaplan–Meier implant survivorship were compared between groups.

**Results:**

The CR group demonstrated greater preoperative varus alignment (median robotic hip–knee–ankle angle [rHKA] 5° vs. 3°, *p* = 0.01). Compared with PS implants, CR inserts were implanted with greater tibial varus (2° vs. 1°, *p* < 0.0001), greater posterior tibial slope (3° vs. 1°, *p* < 0.0001) and slightly greater femoral external rotation (*p* = 0.009). Polyethylene inserts >9 mm were required more frequently in the PS group (39.0% vs. 15.5%, *p* < 0.0001). At final follow‐up, KSS knee (*p* = 0.56), KSS function (*p* = 0.62) and FJS (*p* = 0.06) were comparable between groups. No statistically significant difference in survivorship was detected for aseptic loosening (0.68% vs. 0.74%, *p* = 0.95) and all‐cause revision (2.03% vs. 1.47%, *p* = 0.72). The DyAK classification changed significantly more frequently in the PS group than in the CR group (69.9% vs. 52.7%, *p* = 0.003), despite similar postoperative clinical outcomes.

**Conclusion:**

CR and PS polyethylene inserts showed no significant differences in final clinical scores or implant survivorship at a minimum follow‐up of 3 years but demonstrated different patterns of postoperative dynamic alignment, suggesting that the DyAK classification may provide complementary information beyond conventional outcome measures.

**Level of Evidence:**

Level III.

## Graphical abstract

CR and PS inserts showed no significant differences in final clinical scores or survivorship, but different DyAK transition patterns after robotic‐assisted TKA.

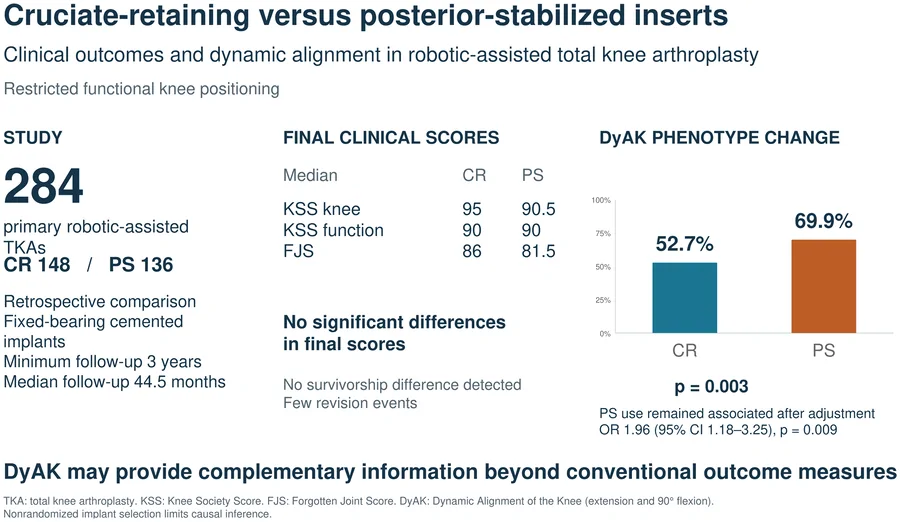

**Abbreviations**
BMI: body mass index
CI: confidence interval
CR: cruciate‐retaining
CS: cruciate substituting
CT: computed tomography
DAIR: debridement, antibiotics and implant retention
DyAK: Dynamic Alignment of the Knee
FA: functional alignment
FJS: Forgotten Joint Score
HR: hazard ratio
IQR: interquartile range
KSS: Knee Society Score
PCL: posterior cruciate ligament
PS: posterior‐stabilized
rHKA: robotic hip–knee–ankle angle
TEA: transepicondylar axis
TKA: total knee arthroplasty

## INTRODUCTION

Functional alignment (FA) has emerged as a personalized alignment philosophy in total knee arthroplasty (TKA), aiming to restore each patient's constitutional anatomy and soft tissue envelope rather than forcing all knees to a neutral mechanical target [3, 13, 23]. By working within predefined coronal and sagittal boundaries while balancing the knee according to its native ligament behaviour, FA seeks to reproduce more physiological knee kinematics [16, 45]. Computed tomography (CT)‐based robotic platforms have proven safe and accurate, facilitating the clinical adoption of this philosophy by enabling three‐dimensional planning, real‐time assessment of ligament balance and precise implant positioning [6, 11, 12, 26, 40, 50]. Although early and mid‐term studies have demonstrated excellent clinical outcomes and reproducible implant positioning, FA remains a relatively recent concept and the influence of several technical variables, including polyethylene insert design, has not yet been adequately investigated [2, 10, 14, 15, 27, 29, 30].

The choice between cruciate‐retaining (CR) and posterior‐stabilized (PS) implants is primarily determined by posterior cruciate ligament (PCL) integrity and surgeon preference [35, 39, 47]. CR designs preserve the native PCL to maintain physiological femoral rollback, whereas PS implants substitute its function through a cam‐post mechanism [19, 32]. Comparative studies have reported conflicting results regarding clinical outcomes, range of motion, complications and implant survivorship, while almost all available evidence originates from mechanically aligned TKA [20, 22, 32, 48, 49, 51]. Consequently, it remains uncertain whether these findings can be extrapolated to FA. Furthermore, previous comparisons have relied almost exclusively on static clinical and radiographic assessment [8, 28]. Recently, the Dynamic Alignment of the Knee (DyAK) classification was introduced to characterize the dynamic coronal behaviour of the knee throughout the range of motion, providing a novel framework for evaluating postoperative knee mechanics beyond conventional static alignment classifications [4]. In its initial description, preservation of the DyAK type was associated with greater improvement in KSS knee than changes in both extension and flexion, despite comparable final clinical outcomes [4]. Thus, DyAK may capture clinically relevant changes not evident on static assessment, although its relationship with patient outcomes and implant survivorship requires further validation.

The purpose of this retrospective comparative study was to compare CR and PS implants in robotic‐assisted TKA performed according to FA principles, with a minimum follow‐up of 3 years, with respect to clinical outcomes, implant survivorship, implant positioning and dynamic knee behaviour according to the DyAK classification. It was hypothesized that CR and PS implants would demonstrate comparable clinical outcomes and survivorship, but could be associated with different postoperative DyAK patterns because of their differences in PCL preservation and flexion‐gap mechanics.

## METHODS

### Study design

This retrospective comparative study utilized a prospectively maintained database. The analysis included all adult patients who underwent primary robotic‐assisted TKA at the 2nd Department of Orthopaedic Surgery of Hygeia Hospital, Athens, Greece, between June 2020 and June 2023 under the principles of FA using the image‐based robotic system (Mako, Stryker, Mako Surgical Corp.). All patients received a fixed‐bearing cemented Triathlon implant (Stryker), either with a CR or PS polyethylene insert. CR inserts were used in patients with an intact and functional PCL, defined intraoperatively by preserved ligament continuity and adequate tension and flexion stability during trial assessment. PS inserts were selected in cases of PCL insufficiency, significant flexion contracture or when additional posterior stability was considered necessary based on the preoperative clinical and intraoperative evaluation. This assessment followed the same intraoperative sequence in all cases.

Patients with previous osteotomies, fractures, ligament deficiencies preventing the application of FA principles, inflammatory arthritis or inadequate follow‐up (<3 years) were excluded. Patients undergoing revision arthroplasty as the index procedure were excluded; however, patients who underwent reoperation or revision after the primary TKA remained in the cohort regardless of when the event occurred. There was no exclusion criterion based on the degree of coronal deformity.

### Surgical technique

All procedures were performed using the Mako robotic‐arm‐assisted system (Stryker, Mako Surgical Corp.) with preoperative CT‐based planning. A restricted functional knee positioning strategy was applied in all cases [25]. Tibial coronal alignment was planned within 3° of varus and 2° of valgus, while femoral coronal alignment was maintained within 6° of valgus and 3° of varus. Tibial posterior slope was individualized according to implant design, with a target of 0°–3° for CR implants and 0°–1° for PS implants. Femoral rotation was referenced to the transepicondylar axis (TEA) and adjusted according to the native trochlear morphology. Implant positioning was dynamically refined after osteophyte removal by assessing mediolateral gaps in extension and at 90° of flexion. A mildly asymmetrical gap pattern was targeted, with a balanced medial compartment and approximately 1–2 mm of residual lateral laxity in both extension and flexion, while soft‐tissue releases were minimized whenever possible. The anterior compartment was optimized by matching the femoral implant trochlea to the native trochlear anatomy, with adjustments in femoral sizing and positioning performed when required to optimize patellofemoral tracking [31]. The patella was not resurfaced; circumferential denervation and osteophyte removal were performed in all cases.

### Clinical and intraoperative evaluation

Patient demographics, including age, sex, body mass index (BMI) and duration of follow‐up were recorded.

Clinical evaluation consisted of the Knee Society Score (KSS) knee and function components preoperatively and at the final follow‐up. Forgotten Joint Score (FJS) was assessed at final follow‐up.

Intraoperative robotic data were extracted directly from the robotic platform. Preoperative coronal alignment was recorded in extension and at 90° of knee flexion, as the robotic hip–knee–ankle angle (rHKA) before bone resections. Implant positioning data included tibial coronal alignment (varus/valgus) and posterior slope, measured relative to the mechanical axis of the tibia and femoral coronal alignment (varus/valgus) and flexion, measured relative to the mechanical axis of the femur. Femoral component rotation was referenced to the surgical TEA. In addition, medial and lateral tibial resection thickness, distal and posterior femoral resection thickness, polyethylene insert thickness and postoperative rHKA after implantation (in extension and at 90° of knee flexion) were recorded.

### DyAK

Dynamic knee behaviour was evaluated using the DyAK classification [4]. DyAK is based on robotic measurements of the hip–knee–ankle angle obtained in full extension (rHKA‐E) and at 90° of flexion (rHKA‐90F), without the application of varus‐valgus stress. Both measurements are categorized as varus (<−3°), neutral (−3° to 3°) or valgus (>3°), generating a 3 × 3 matrix comprising nine dynamic knee phenotypes (D1–D9). The DyAK type was determined both before bone resections (preoperative assessment) and after final implantation. The proportion of knees maintaining or changing the preoperative DyAK classification between the two assessments was compared between the CR and PS groups.

For descriptive and statistical analyses, rHKA values were retained according to the robotic‐system convention, with varus recorded as positive and valgus as negative. For DyAK classification and graphical presentation, the signs were inverted to follow the published DyAK convention, in which varus is negative and valgus is positive.

### Implants' survivorship

All postoperative revisions were reviewed. Survival time was calculated from the date of the index TKA to the date of revision. Patients revised before 3 years remained included and were counted as events at the date of revision. Implant survivorship for aseptic loosening and all‐cause revision was evaluated using Kaplan‐Meier survival analysis.

### Statistical analysis

The Kolmogorov–Smirnov test was used to assess data normality. Continuous variables were compared using either the independent‐samples *t* test or the Mann–Whitney *U* test according to data distribution. For nonparametric clinical‐outcome comparisons, effect estimates were additionally reported as Hodges–Lehmann median differences with 95% confidence intervals (CIs). Differences were calculated as PS minus CR for insert‐group comparisons and as DyAK changed minus retained for phenotype‐transition comparisons. Changes in KSS knee and KSS function were calculated as postoperative minus preoperative scores and compared between the CR and PS groups. An exploratory analysis compared changes in KSS knee and KSS function and postoperative FJS between knees that retained and those that changed their DyAK type. The proportions of patients achieving the minimal clinically important difference (MCID) were also compared between the CR and PS groups. MCID achievement was defined as an improvement of at least 6.6 points for KSS knee and 6.3 points for KSS function, based on previously published thresholds [7, 21, 34]. Categorical variables, including complication rates, MCID achievement and DyAK classification frequencies, were compared using the *χ* ^2^ test. Implant survivorship was evaluated using Kaplan–Meier survival analysis with hazard ratios (HRs) and 95% CIs. Statistical significance was defined as *p* < 0.05. Because this was a retrospective consecutive‐case (‘all‐comers’) cohort comprising all eligible procedures during the predefined study period, no a priori sample size calculation was performed. A post hoc sensitivity analysis was subsequently conducted to assess whether the available cohort was sufficient to detect between‐group differences corresponding to the published MCID thresholds, assuming a two‐sided *α* of 0.05, 80% power and the observed group standard deviations.

To account for potential confounding by baseline characteristics and preoperative knee morphology, a multivariable binary logistic regression analysis was performed to evaluate whether insert design was independently associated with a change in DyAK type. DyAK type change (yes/no) was entered as the dependent variable, while insert design, age, sex, BMI, preoperative rHKA in extension and preoperative rHKA at 90° of flexion were entered simultaneously as independent variables. The analysis included patients with complete data for all covariates (*n* = 283), and adjusted odds ratios (ORs) with 95% CIs were calculated.

All analyses were performed using MedCalc® Statistical Software Version 23.5.2 (MedCalc Software Ltd).

## RESULTS

### Preoperative characteristics

A total of 284 patients were included in the study, comprising 148 patients in the CR group and 136 in the PS group (Figure 1). The median follow‐up was 44.5 months (interquartile range [IQR]: 36–57), with a minimum follow‐up of 3 years. There were no significant differences in age, BMI or preoperative clinical scores between the two groups. The CR group demonstrated greater preoperative varus alignment according to rHKA (5° [IQR: 2–9] vs. 3° [IQR: −3 to 10], *p *= 0.01) (Table 1).

**Figure 1:**
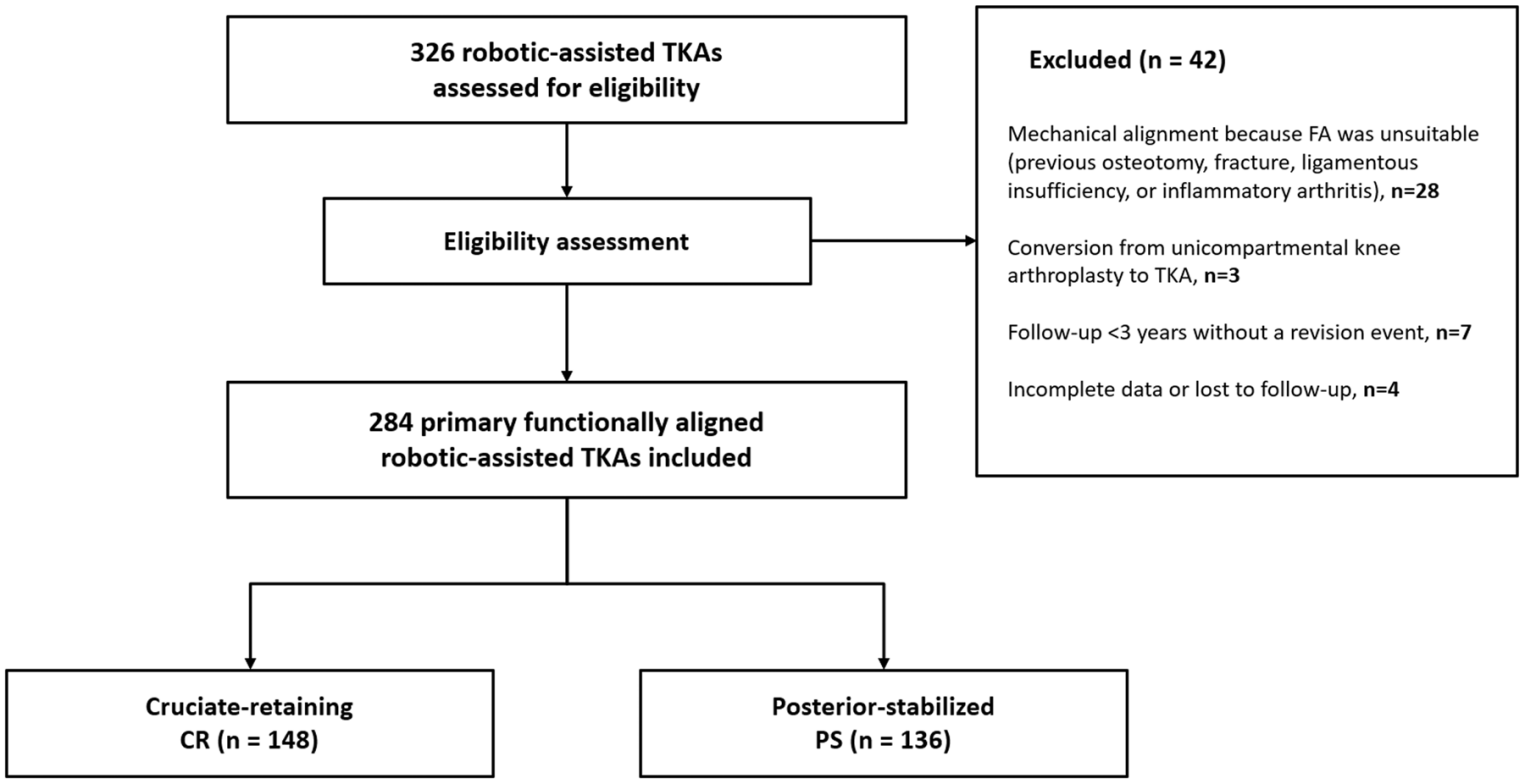
Flow diagram of patient selection. The minimum 3‐year follow‐up was calculated from the index primary TKA, rather than from any subsequent reoperation. Patients who underwent reoperation or revision within 3 years remained included in the cohort. CR, cruciate‐retaining; FA, functional alignment; PS, posterior‐stabilized; TKA, total knee arthroplasty.

**Table 1:** Comparison of preoperative demographic, clinical and intraoperative robotic characteristics between the cruciate‐retaining (CR) and posterior‐stabilized (PS) groups.

	CR group (*N* = 148)	PS group (*N* = 136)	*p* value
Demographics			
Age (years)	73 (IQR 68–77)	73 (IQR 68–76)	0.83
Female gender	51.33% (*N* = 76)	53.68% (*N* = 73)	0.69
BMI (kg/m^2^)	28.5 (IQR 24.8–32.73)	27.68 (IQR 24.39–32.46)	0.81
Preoperative clinical evaluation			
KSS knee	64 (IQR 55–73)	69 (IQR 57–77)	0.06
KSS function	70 (IQR 57.5–76.5)	70 (IQR 60–80)	0.06
Intraoperative assessment with the Robotic system			
Alignment (varus) rHKA	5 (IQR 2–9)	3 (IQR −3 to 10)	**0.01**

*Note*: Continuous variables are presented as median (IQR) and categorical variables as number (%). Alignment (degrees in varus) was determined intraoperatively using the rHKA. Bold values indicate statistical significance (*p* < 0.05). rHKA values are presented according to the robotic‐system convention: positive values indicate varus and negative values indicate valgus.

Abbreviations: BMI, body mass index; IQR, interquartile range; KSS, Knee Society Score; rHKA, robotic hip–knee–ankle angle; *N*, number.

### Implant positioning

Implant positioning differed between the two insert designs. The CR group demonstrated greater tibial varus (2° [IQR: 1–2] vs. 1° [IQR: 0–1], *p* < 0.0001), greater posterior tibial slope (3° [IQR: 3–3] vs. 1° [IQR: 1–1], *p* < 0.0001) and slightly greater femoral external rotation (1° [IQR: 0.1–2] vs. 0.8° [IQR: 0–1.9], *p *= 0.009) (Table 2).

**Table 2:** Comparison of femoral and tibial implant positioning between the cruciate‐retaining (CR) and posterior‐stabilized (PS) groups during robotic‐assisted total knee arthroplasty.

	CR group (*N* = 148)	PS group (*N* = 136)	*p* value
Tibial implant positioning (degrees with reference to the tibial mechanical axis)			
Varus	2 (IQR 1 to 2)	1 (IQR 0 to 1)	**<0.0001**
Posterior slope	3 (IQR 3 to 3)	1 (IQR 1 to 1)	**<0.0001**
Femoral implant positioning (degrees with reference to the femoral mechanical axis)			
External rotation (reference TEA)	1 (IQR 0.1 to 2)	0.8 (IQR 0 to 1.9)	**0.009**
Valgus	0.5 (IQR 0 to 1)	1 (IQR 0 to 2)	0.23
Flexion	4.95 (IQR 3 to 5)	4.5 (IQR 3 to 5)	0.38

*Note*: Values are presented as median (IQR). Tibial coronal alignment and posterior slope are referenced to the tibial mechanical axis, whereas femoral coronal alignment and flexion are referenced to the femoral mechanical axis. Femoral rotation is referenced to the surgical TEA. Bold values indicate statistical significance (*p* < 0.05).

Abbreviations: IQR, interquartile range; TEA, surgical transepicondylar axis.

Bone resection thicknesses were largely comparable between groups. However, the CR group required a greater lateral tibial resection (7 [IQR: 7–8] vs. 7 [IQR: 6–8] mm, *p* = 0.02] and a greater posterior lateral femoral resection (6.5 [IQR: 5.5–8] vs. 6 [IQR: 5–7] mm, *p *= 0.004) (Table 3).

**Table 3:** Comparison of tibial and femoral bone resection thicknesses between the cruciate‐retaining (CR) and posterior‐stabilized (PS) groups.

	CR group (*N* = 148)	PS group (*N* = 136)	*p* value
Tibial cuts (mm)			
Medial	5 (IQR 4–5.5)	5 (IQR 4–5.5)	0.52
Lateral	7 (IQR 7–8)	7 (IQR 6–8)	**0.02**
Femoral cuts (mm)			
Distal medial	8.5 (IQR 8–9)	8.5 (IQR 8–9)	0.87
Distal lateral	5.25 (IQR 4–6.5)	5 (IQR 4–6)	0.24
Posterior medial	9.5 (IQR 9–10.5)	9.5 (IQR 8.5–10.5)	0.26
Posterior lateral	6.5 (IQR 5.5–8)	6 (IQR 5–7)	**0.004**

*Note*: Values are presented as median (IQR) in millimetres (mm). Bold values indicate statistical significance (*p* < 0.05).

Abbreviation: IQR, interquartile range.

Polyethylene inserts thicker than 9 mm were used more frequently in the PS group (38.97% vs. 15.54%, *p* < 0.0001).

### Postoperative outcomes

At final follow‐up, postoperative KSS knee and KSS function did not differ significantly between the CR and PS groups (*p* = 0.56 and *p* = 0.62, respectively). FJS was numerically higher in the CR group, although the Hodges–Lehmann difference for PS − CR was −4.0 points (95% CI: −8.0 to 0.0; *p* = 0.06). Improvement in KSS knee was slightly greater in the CR group (Hodges–Lehmann difference, PS − CR: −3.0 points [95% CI: −7.0 to 0.0]; *p* = 0.048), whereas improvement in KSS function did not differ significantly (*p* = 0.289). MCID achievement was comparable between groups for KSS knee (93.2% vs. 91.9%; *p* = 0.669) and KSS function (89.2% vs. 86.8%; *p* = 0.528). For 80% power, the sensitivity analysis required 112 CR and 103 PS knees for KSS knee, 140 and 129 for KSS function, and 29 and 27 for FJS, respectively; the available sample sizes exceeded these requirements for all three outcomes.

The CR group demonstrated slightly greater postoperative rHKA (*p *= 0.008) (Table 4).

**Table 4:** Comparison of postoperative clinical outcomes and intraoperative robotic alignment measurements between the cruciate‐retaining (CR) and posterior‐stabilized (PS) groups.

	CR group (*N* = 148)	PS group (*N* = 136)	Hodges–Lehmann difference, PS − CR (95% CI)	*p* value
Postoperative clinical evaluation				
KSS knee	95 (IQR 90–100)	90.5 (IQR 90–100)	0.0 (0.0–0.0)	0.56
KSS function	90 (IQR 90–100)	90 (IQR 90–100)	0.0 (0.0–0.0)	0.62
FJS	86 (IQR 74.5–94)	81.5 (IQR 65–92)	−4.0 (−8.0 to 0.0)	0.06
ΔKSS knee	29 (IQR 20–40)	24 (IQR 17–36.5)	−3.0 (−7.0 to 0.0)	**0.048**
ΔKSS function	25.5 (IQR 12–40)	22 (IQR 15–30)	0.0 (−5.0 to 0.0)	0.29
KSS knee MCID achieved, *n* (%)	138 (93.2%)	125 (91.9%)	—	0.67
KSS function MCID achieved, *n* (%)	132 (89.2%)	118 (86.8%)	—	0.528
Assessment from the robotic system				
Postoperative Mako alignment (varus degrees) rHKA	3 (IQR 1–4)	2 (IQR 0–4)	***—***	**0.008**

*Note*: Continuous variables are presented as median (IQR). For the postoperative clinical outcomes, effect estimates are presented as Hodges–Lehmann median differences with 95% confidence intervals (CIs), calculated as PS minus CR. The Hodges–Lehmann estimate represents the location shift between distributions and may differ from the arithmetic difference between group medians. ΔKSS was calculated as the postoperative minus preoperative score. MCID achievement was defined as an improvement of at least 6.6 points for KSS knee and 6.3 points for KSS function. Postoperative alignment was determined using the rHKA. Bold values indicate statistical significance (*p* < 0.05). rHKA values are presented according to the robotic‐system convention: positive values indicate varus and negative values indicate valgus.

Abbreviations: FJS, Forgotten Joint Score; IQR, interquartile range; KSS, Knee Society Score; rHKA, robotic hip–knee–ankle angle.

### DyAK

The distribution of preoperative DyAK phenotypes differed between the two groups. Type D1 was the most frequent preoperative phenotype in both groups (45.3% in the CR group vs. 34.6% in the PS group, *p* = 0.066). Compared with the CR group, the PS group demonstrated fewer D2 phenotypes (0.7% vs. 10.8%, *p* < 0.001) and higher proportions of D6 (6.6% vs. 0.7%, *p* = 0.007), D8 (14.0% vs. 2.7%, *p* < 0.001) and D9 (12.5% vs. 2.7%, *p* = 0.002).

Following implantation, D5 became the predominant postoperative phenotype in both groups (54.7% in the CR group vs. 62.5% in the PS group, *p* = 0.184). Among the postoperative DyAK categories, only D1 remained significantly more frequent in the CR group (19.6% vs. 10.3%, *p* = 0.029), whereas the remaining phenotypes were comparable between groups (Table 5).

**Table 5:** Comparison of preoperative and postoperative Dynamic Alignment of the Knee (DyAK) phenotypes between the cruciate‐retaining (CR; Group A) and posterior‐stabilized (PS; Group B) groups.

	DyAK	Group A (*n* = 148)	Group B (*n* = 136)	*p* value
Preoperatively	D1	67 (45.3%)	47 (34.6%)	0.066
	D2	16 (10.8%)	1 (0.7%)	**<0.001**
	D3	0 (0.0%)	3 (2.2%)	0.07
	D4	22 (14.9%)	20 (14.7%)	0.96
	D5	34 (23.0%)	20 (14.7%)	0.076
	D6	1 (0.7%)	9 (6.6%)	**0.007**
	D7	0 (0.0%)	0 (0.0%)	1.000
	D8	4 (2.7%)	19 (14.0%)	**<0.001**
	D9	4 (2.7%)	17 (12.5%)	**0.002**
Postoperatively	D1	29 (19.6%)	14 (10.3%)	**0.029**
	D2	13 (8.8%)	8 (5.9%)	0.352
	D3	0 (0.0%)	0 (0.0%)	1.000
	D4	24 (16.2%)	23 (16.9%)	0.874
	D5	81 (54.7%)	85 (62.5%)	0.184
	D6	0 (0.0%)	1 (0.7%)	0.31
	D7	0 (0.0%)	0 (0.0%)	1.000
	D8	1 (0.7%)	5 (3.7%)	0.079
	D9	0 (0.0%)	0 (0.0%)	1

*Note*: Values are presented as *n* (%). DyAK is a dynamic classification derived from robotic hip–knee–ankle angle measurements in full extension and 90° of flexion, generating nine phenotypes (D1–D9). *p* values represent between‐group comparisons for each phenotype. Statistically significant *p* values are shown in bold.

Overall, the DyAK classification changed in 52.7% of knees in the CR group and 69.9% in the PS group (*p *= 0.003) (Figure 2).

**Figure 2:**
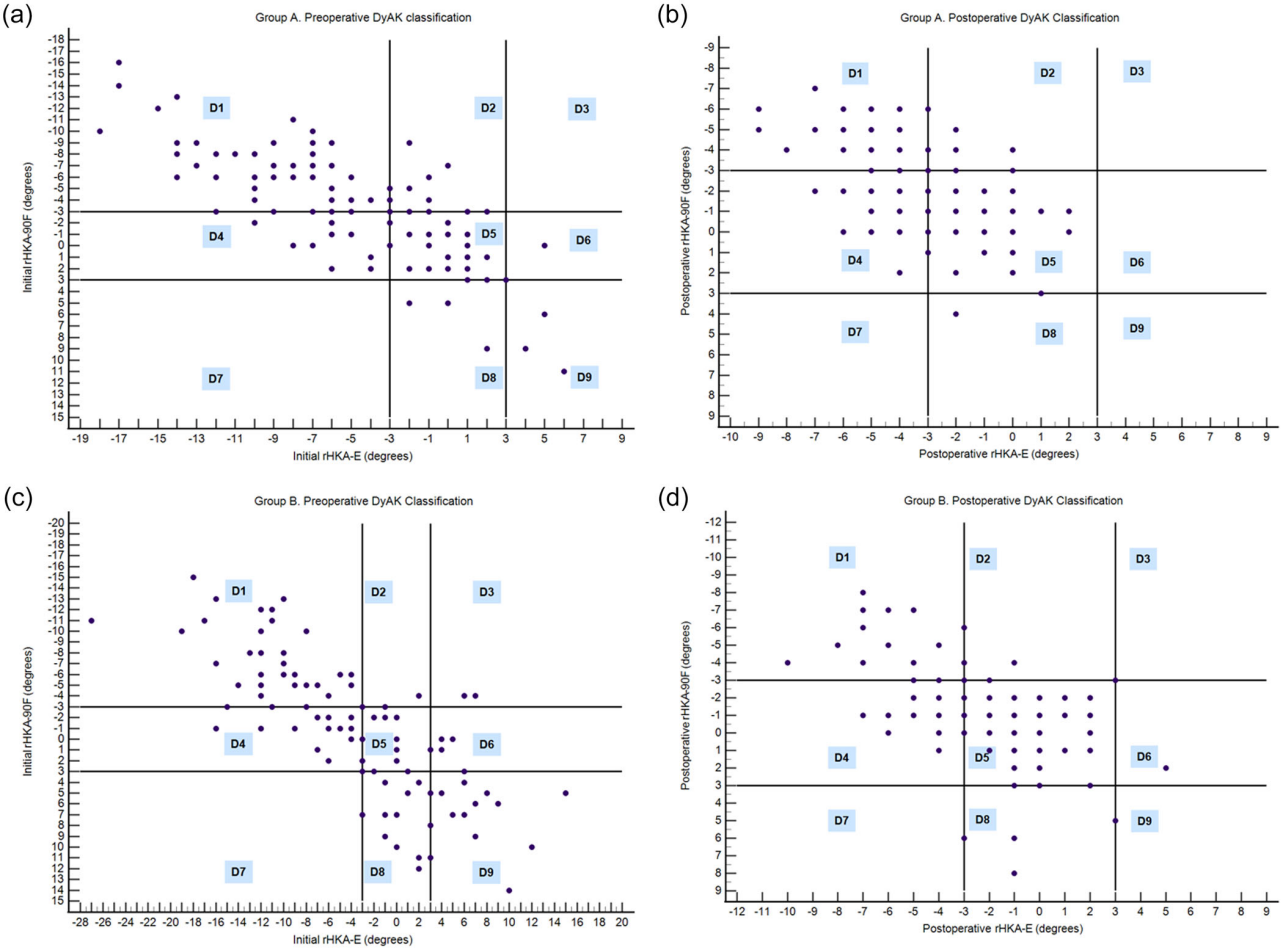
Scatter plots illustrating the distribution of Dynamic Alignment of the Knee (DyAK) phenotypes in the cruciate‐retaining (CR; Group A) and posterior‐stabilized (PS; Group B) groups before and after robotic‐assisted total knee arthroplasty. (a) Preoperative DyAK classification in the CR group. (b) Postoperative DyAK classification in the CR group. (c) Preoperative DyAK classification in the PS group. (d) Postoperative DyAK classification in the PS group. Each dot represents one knee. The horizontal and vertical lines at ±3° define the boundaries between varus, neutral and valgus alignment in flexion (rHKA‐90F) and extension (rHKA‐E), respectively, thereby generating the nine DyAK phenotypes (D1–D9). Negative values represent varus alignment and positive values represent valgus alignment according to the intraoperative robotic hip–knee–ankle angle (rHKA) measurements.

In the multivariable logistic regression analysis (*n* = 283), PS insert use remained independently associated with higher odds of DyAK type change after adjustment for age, sex, BMI and preoperative rHKA in extension and at 90° of flexion (adjusted OR = 1.96; 95% CI: 1.18–3.25; *p* = 0.009). None of the remaining covariates were independently associated with DyAK type change (all *p* > 0.05). The overall model was statistically significant (*p* = 0.024).

Knees retaining the preoperative DyAK type demonstrated greater improvement in KSS knee than those changing type (Hodges–Lehmann difference, changed minus retained: −4.0 points [95% CI: −8.0 to 0.0]; *p *= 0.033). No significant differences were observed in KSS function improvement or postoperative FJS (Table 6).

**Table 6:** Clinical outcomes according to retention or change of the DyAK phenotype.

Outcome	DyAK retained	DyAK changed	Hodges–Lehmann difference (95% CI)^a^	*p* value
ΔKSS knee, points	30 (20–40), *n* = 111	24 (17.75–37), *n* = 173	−4.0 (−8.0 to 0.0)	**0.033**
ΔKSS function, points	20 (10–35), *n* = 111	28 (15–35), *n* = 173	0.0 (0.0–5.0)	0.412
Postoperative FJS, points	86 (66.5–94), *n* = 108	84 (67–92), *n* = 165	0.0 (−4.0 to 2.0)	0.722

*Note*: Values are presented as median (interquartile range). ΔKSS was calculated as the postoperative minus preoperative score.

Abbreviations: CI, confidence interval; DyAK, Dynamic Alignment of the Knee; FJS, Forgotten Joint Score; KSS, Knee Society Score.

^a^ Hodges–Lehmann differences were calculated as DyAK changed minus DyAK retained. Bold values indicate statistical significance (*p* < 0.05).

### Implants' survivorship

Kaplan–Meier survival analysis demonstrated no significant difference in implant survivorship for aseptic loosening between the CR and PS groups (0.68% vs. 0.74%, *p *= 0.95). The CR group did not have a significantly different risk of aseptic loosening compared with the PS group (HR = 0.92; 95% CI: 0.06–14.68) (Figure 3).

**Figure 3:**
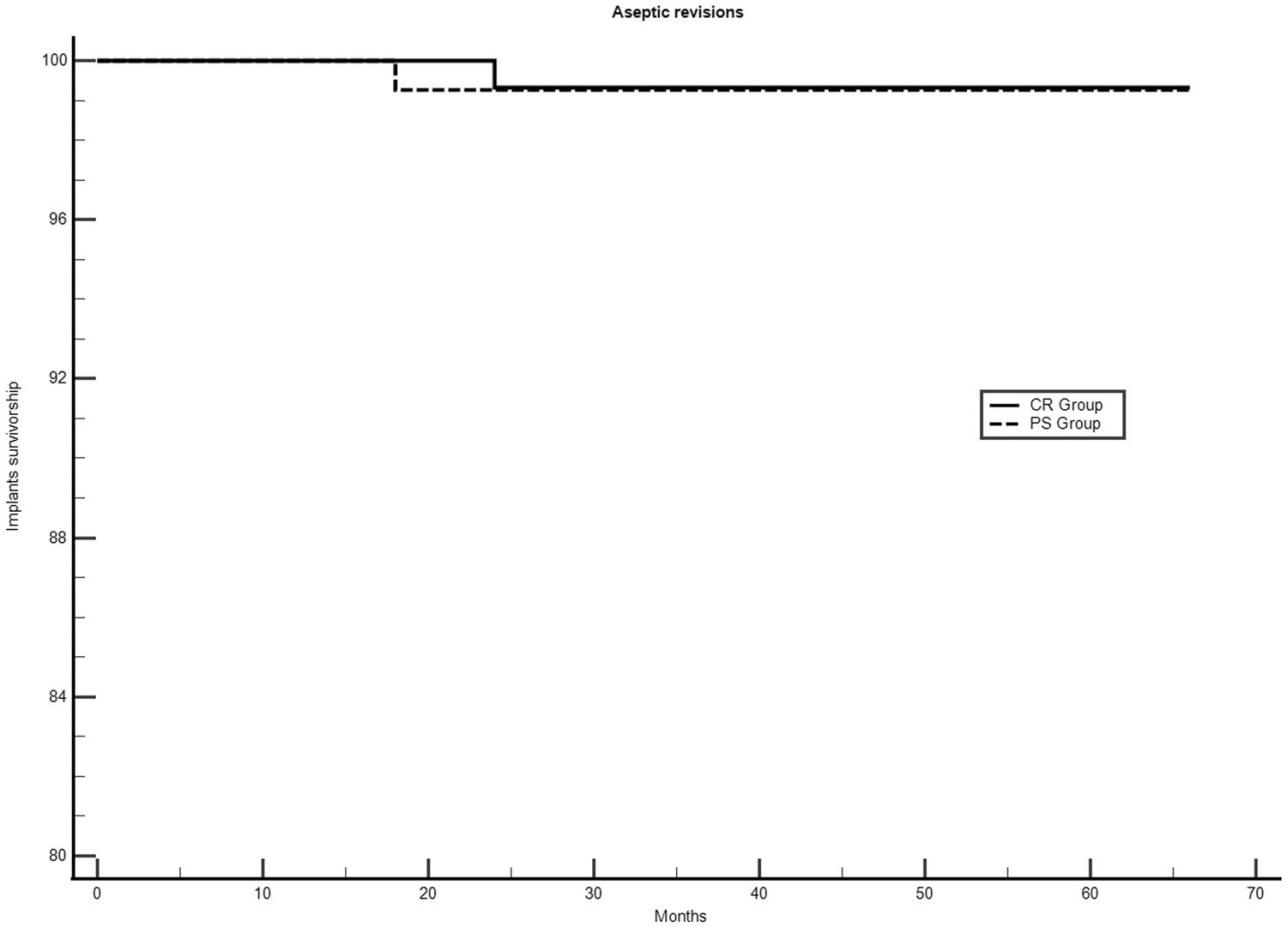
Kaplan–Meier survival analysis comparing implant survivorship for aseptic loosening between the cruciate‐retaining (CR) and posterior‐stabilized (PS) groups. Implant survivorship was comparable between the two groups at a minimum follow‐up of 3 years (*p* = 0.95). The cumulative revision rate for aseptic loosening was 0.68% in the CR group (hazard ratio [HR] = 0.92; 95% confidence interval [CI], 0.06–14.68) and 0.74% in the PS group (HR = 1.09; 95% CI, 0.07–17.51).

Similarly, all‐cause revision rates were comparable between the two groups (2.03% vs. 1.47%, *p *= 0.72). Two debridement, antibiotics and implant retention (DAIR) procedures were performed in the CR group and one in the PS group. Kaplan–Meier survival analysis showed that the CR group did not have a significantly different risk of all‐cause revision compared with the PS group (HR = 1.38; 95% CI: 0.24–7.99) (Figure 4).

**Figure 4:**
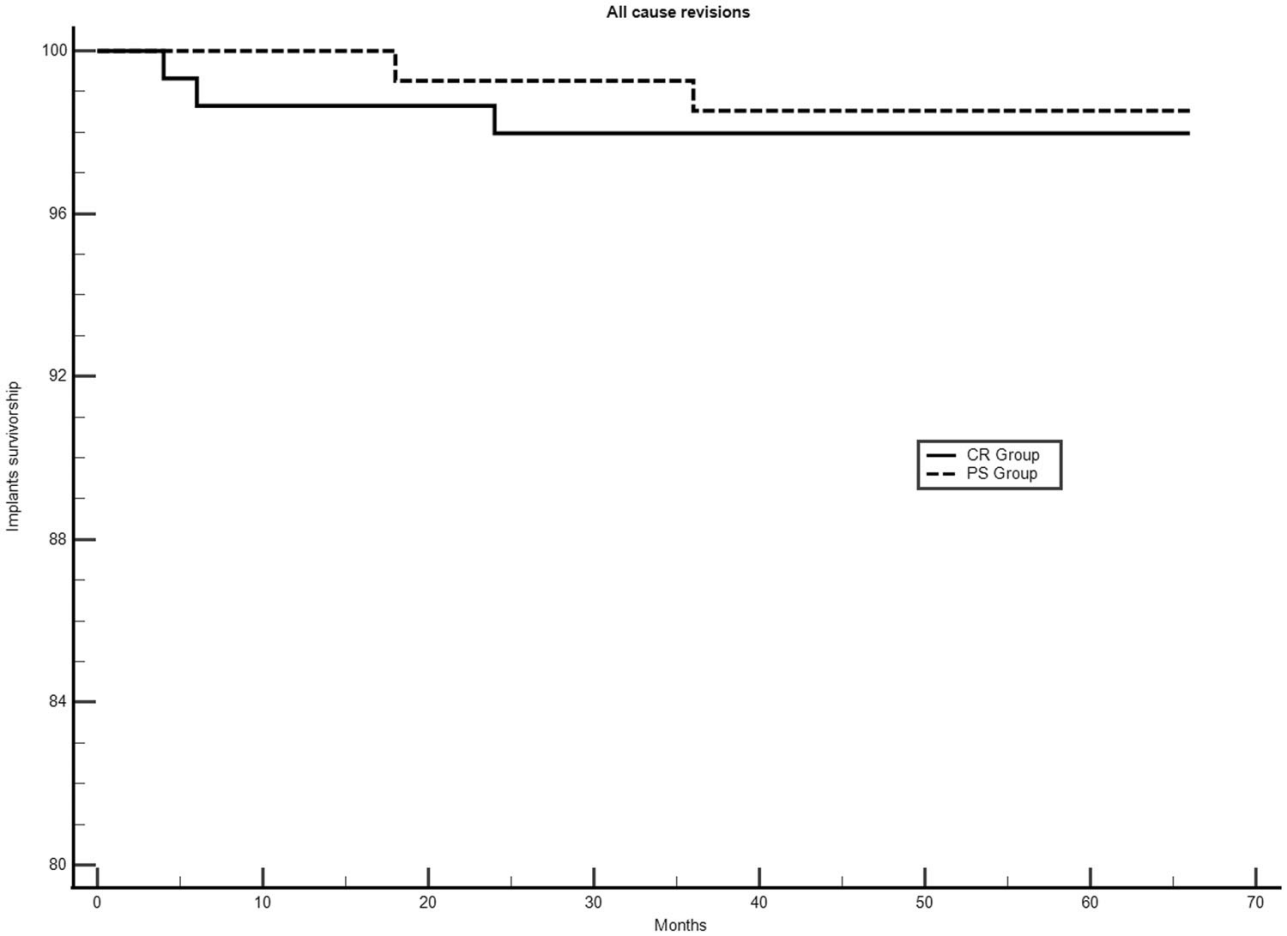
Kaplan–Meier survival analysis of the cruciate‐retaining (CR) and posterior‐stabilized (PS) groups, taking into account all‐cause revision as the endpoint. Survival analysis demonstrated comparable implant survivorship between the two groups at a minimum follow‐up of 3 years (*p* = 0.72). The cumulative all‐cause revision rates were 2.03% for the CR group (two debridement, antibiotics and implant retention [DAIR] procedures and one aseptic revision) and 1.47% for the PS group (one DAIR procedure and one aseptic revision).

## DISCUSSION

The most important finding of the present study was that CR and PS polyethylene inserts provided comparable clinical outcomes and implant survivorship at a minimum follow‐up of 3 years when implanted according to the principles of FA. These findings should be interpreted in the context of the different preoperative morphologies, with greater varus alignment in the CR group and a higher prevalence of valgus DyAK phenotypes in the PS group. Differences were also observed in implant positioning and intraoperative planning, with CR knees demonstrating greater tibial varus, as well as greater posterior tibial slope in accordance with the predefined implant‐specific targets, whereas PS knees more frequently required thicker polyethylene inserts. Finally, the DyAK classification demonstrated a significantly higher rate of postoperative phenotype change in the PS group. This association persisted after adjustment for age, sex, BMI and preoperative coronal alignment in extension and flexion; however, residual confounding related to PCL status, flexion contracture and implant selection prevents causal attribution to polyethylene design alone.

The present study demonstrated a significantly greater preoperative varus deformity in the CR group, whereas knees treated with PS implants were relatively more valgus. This finding most likely reflects the implant selection algorithm rather than differences in the study population. Although both implant designs can be successfully used in a wide spectrum of coronal deformities, PS implants are frequently preferred in knees with valgus deformity, flexion contracture or when PCL competence and flexion balancing are uncertain, as sacrificing the PCL and using a cam‐post mechanism may facilitate soft tissue balancing and improve stability [9, 42, 44, 52].

Although both implant designs were implanted according to the same FA protocol, significant differences in implant positioning and intraoperative planning were observed. The CR group demonstrated greater tibial varus and posterior slope, whereas PS implants more frequently required thicker polyethylene inserts. The difference in posterior tibial slope was inherent to the surgical protocol, which specified targets of 0°–3° for CR and 0°–1° for PS implants and should not be interpreted as an independent effect of insert design. The greater tibial varus observed in the CR group is consistent with the principles of functional knee positioning, where correction of constitutional varus is preferentially achieved from the tibial side while remaining within predefined safe boundaries [24]. Interestingly, the CR group demonstrated greater lateral tibial and posterolateral femoral resection thicknesses. These differences likely reflect the distinct three‐dimensional implant positioning strategy associated with CR inserts, which in the present study was characterized by greater tibial varus, posterior slope and femoral external rotation. Consequently, the observed differences in bone resection thicknesses most likely represent the geometric effect of implant orientation required to achieve equivalent mediolateral soft tissue balance while preserving the PCL, rather than a different surgical philosophy. Similarly, thicker polyethylene inserts were more frequently required in the PS group, likely reflecting the combined effect of the relatively more valgus morphology, the altered flexion‐gap characteristics following PCL substitution and the implant positioning necessary to achieve balanced flexion and extension gaps [1, 37, 38, 43]. Although several implant‐positioning and resection variables reached statistical significance, the absolute between‐group differences were small. Despite the high measurement accuracy of the robotic platform, the median difference in femoral rotation was only 0.2°, while differences in bone‐resection thickness were generally ≤1 mm. The clinical relevance of differences of this magnitude remains uncertain, particularly in the absence of corresponding differences in clinical outcomes. Overall, these findings describe how CR and PS inserts were selected and positioned within the predefined FA workflow and should not be interpreted as intrinsic or causal differences between the implant designs.

Postoperative clinical outcomes were comparable between the CR and PS groups, with no significant differences in KSS knee, KSS function or FJS at final follow‐up. Although improvement in KSS knee was statistically greater in the CR group, the estimated difference was modest (Hodges–Lehmann difference for PS − CR: −3.0 points; 95% CI: −7.0 to 0.0), and the proportion achieving MCID was comparable between groups. Improvement in KSS function and MCID achievement for KSS function were also comparable. Moreover, FJS was numerically higher in the CR group; however, the Hodges–Lehmann estimate for PS − CR was −4.0 points (95% CI: −8.0 to 0.0), consistent with the absence of a statistically significant between‐group difference. The sensitivity analysis showed that the available cohort exceeded the sample sizes required to detect MCID‐sized differences in KSS knee, KSS function and FJS with 80% power, supporting the adequacy of the cohort for detecting clinically meaningful differences in these outcomes.

These findings are consistent with the limited available literature on insert design in functionally aligned robotic‐assisted TKA [8, 28]. Daffara et al. compared 84 CR and 80 PS robotic‐assisted TKAs with a minimum 2‐year follow‐up and reported no significant differences in FJS‐12, KOOS‐JR or satisfaction scores between the two designs [8]. Similarly, a previous study comparing cruciate‐substituting (CS) inserts, which preserve the PCL while incorporating increased anterior conformity, and PS inserts under FA principles found comparable KSS, FJS, ROM, complication rates and implant survivorship at a median follow‐up of 36 months [28]. Although not equivalent to CR designs, CS inserts provide related evidence regarding PCL preservation under FA principles. The present study expands on these findings by specifically comparing CR and PS inserts in a larger cohort with a minimum 3‐year follow‐up. Despite the modest difference in KSS knee improvement, final clinical outcomes and MCID achievement were broadly comparable between the groups. These findings should not be interpreted as demonstrating interchangeability or suggesting that implant choice is clinically irrelevant, as selection remains dependent on PCL status, patient‐specific anatomy and intraoperative balancing. Furthermore, no statistically significant difference in implant survivorship was detected between the CR and PS groups, both for aseptic loosening and all‐cause revision. These findings are consistent with those of Daffara et al., who reported only three revisions among 164 robotic‐assisted TKAs without differences between the two insert designs, as well as with Koutserimpas et al. demonstrating equivalent Kaplan‐Meier survivorship between CS and PS inserts under FA principles [8, 28]. Beyond FA, numerous comparative studies and meta‐analyses performed predominantly under mechanical alignment have likewise failed to demonstrate a survivorship advantage of either CR or PS implants at short‐ and mid‐term follow‐up [20, 32, 33, 39, 41, 51]. At the current follow‐up, these findings support selecting the polyethylene insert primarily according to patient‐specific factors, PCL status and intraoperative balancing. However, given the small number of revision events and wide CIs, the survivorship findings should be interpreted cautiously and confirmed in larger cohorts with longer follow‐up.

The DyAK analysis provided the most novel findings of the present study. Although no significant differences in clinical outcomes were observed between the CR and PS groups, the PS group demonstrated a significantly higher rate of postoperative DyAK transition. This finding should be interpreted in the context of the different preoperative knee morphologies. The PS cohort contained a significantly greater proportion of D9 knees, representing valgus alignment in both extension and flexion. Under the principles of FA, valgus morphotypes are generally restored toward a neutral limb alignment while maintaining balanced flexion and extension gaps within predefined safe boundaries, whereas mild constitutional varus is more commonly preserved [5, 17, 18, 36, 46]. Consequently, the greater proportion of valgus phenotypes in the PS group would be expected to undergo a higher rate of DyAK phenotype transition, most commonly toward the neutral D5 phenotype. However, PS insert use remained associated with higher odds of DyAK type change after adjustment for demographic characteristics and preoperative rHKA in both extension and flexion, suggesting that measured baseline alignment did not fully explain this difference. This association should not be interpreted as causal, as residual confounding related to PCL integrity, flexion contracture and intraoperative implant selection cannot be excluded. The potential relevance of DyAK lies in capturing coronal alignment behaviour between extension and flexion that is not represented by static radiographs or extension measurements alone, thereby providing a framework for studying postoperative knee mechanics. In the present study, DyAK identified different patterns of postoperative dynamic alignment despite the absence of significant differences in postoperative KSS and FJS between the groups. When outcomes were analysed according to DyAK retention, knees maintaining the preoperative phenotype demonstrated greater improvement in KSS knee than those changing phenotype, consistent with the initial description of the classification [4]. However, the estimated difference was modest and was not accompanied by differences in KSS function improvement or postoperative FJS. Individual DyAK transitions cannot currently be interpreted as either improvement or deterioration. Until specific phenotypes or transitions are shown to predict function, satisfaction, instability or revision, DyAK should be regarded as a descriptive and research‐oriented classification rather than a validated surgical target or clinical decision‐making tool.

This study has some limitations. First, this was a retrospective, single‐centre comparative study, which is inherently subject to selection bias despite the use of a prospectively maintained database. Second, although this study represents the largest comparative series of CR and PS inserts performed under restricted functional knee positioning with a minimum follow‐up of 3 years, longer‐term follow‐up is required to determine whether the observed clinical findings and survivorship estimates are maintained over time. Although no a priori calculation determined the cohort size, the post hoc sensitivity analysis indicated that the available sample exceeded the requirements for detecting MCID‐sized differences in KSS knee, KSS function and FJS. Nevertheless, smaller between‐group differences cannot be excluded. The small number of revision events limited the statistical power and precision of the survivorship analyses, as reflected by the wide CIs around the HR estimates. Third, implant selection was not randomized but based on PCL status and intraoperative assessment, reflecting routine clinical practice. Although a standardized assessment sequence was followed, PCL competence was determined clinically without a validated quantitative threshold and therefore remained partly surgeon‐dependent. Consequently, the PS group included a greater proportion of knees with valgus morphology, which may have influenced the observed differences in implant positioning and DyAK classification. Although the multivariable analysis adjusted for measured demographic and alignment variables, residual confounding by indication remains possible, while some positioning differences, particularly posterior tibial slope, were protocol‐driven rather than intrinsic to insert design. Finally, DyAK is a recently introduced classification without external validation, and its clinical significance, test–retest reliability, measurement error and minimal detectable change remain undetermined. The ±3° thresholds, applied to both initial and final measurements, were derived from the final postoperative rHKA distributions of the original cohort. Therefore, transitions involving measurements close to these boundaries should be interpreted cautiously. These parameters could not be assessed from the single robotic measurement available at each assessment and require dedicated prospective evaluation.

## CONCLUSION

CR and PS polyethylene inserts showed no significant differences in clinical outcomes at a minimum follow‐up of 3 years when used in robotic‐assisted TKA performed according to restricted functional knee positioning principles. The DyAK classification identified different postoperative phenotype‐transition patterns between the two insert designs. The clinical significance of these transitions remains uncertain and warrants further investigation.

## AUTHOR CONTRIBUTIONS

*Conceptualization*: Christos Koutserimpas, Sébastien Lustig and Konstantinos Dretakis. *Data curation*: Christos Koutserimpas and Enejd Veizi. *Formal analysis*: Christos Koutserimpas and Enejd Veizi. *Investigation*: Christos Koutserimpas, Enejd Veizi, Konstantinos Dretakis and Sébastien Lustig. *Methodology*: Christos Koutserimpas and Enejd Veizi. *Project administration*: Sébastien Lustig and Konstantinos Dretakis. *Supervision*: Sébastien Lustig and Konstantinos Dretakis. *Validation*: Christos Koutserimpas and Konstantinos Dretakis. *Visualization*: Christos Koutserimpas, Enejd Veizi and Sébastien Lustig. *Writing—original draft*: Christos Koutserimpas and Enejd Veizi. *Writing—review and editing*: Sébastien Lustig and Konstantinos Dretakis.

## FUNDING INFORMATION

The authors have no funding to report.

## CONFLICT OF INTEREST STATEMENT

Christos Koutserimpas is a consultant for Smith & Nephew. Sébastien Lustig and Konstantinos Dretakis are consultants for Stryker. The remaining author declares no conflict of interest.

## ETHICS STATEMENT

All procedures were performed in accordance with the ethical standards of the institutional and/or national research committee, the 1964 Helsinki Declaration, later amendments or comparable ethical standards. Approval was granted by the Scientific Committee of Hygeia Hospital, Athens (Reference No. 663, 20 December 2023). As per institutional standards, formal patient consent is not required for this type of study.

## Data Availability

The data that support the findings of this study are available from the corresponding author, upon reasonable request.
